# Dual Effect of *Chrysanthemum indicum* Extract to Stimulate Osteoblast Differentiation and Inhibit Osteoclast Formation and Resorption *In Vitro*

**DOI:** 10.1155/2014/176049

**Published:** 2014-10-28

**Authors:** Jong Min Baek, Ju-Young Kim, Yoon-Hee Cheon, Sun-Hyang Park, Sung-Jun Ahn, Kwon-Ha Yoon, Jaemin Oh, Myeung Su Lee

**Affiliations:** ^1^Department of Anatomy, School of Medicine, Wonkwang University, 344-2 Sinyong-dong, Iksan, Jeonbuk 570-749, Republic of Korea; ^2^BK21plus Program & Department of Smart Life-Care Convergence, Graduate School, Wonkwang University, Iksan,Jeonbuk 570-749, Republic of Korea; ^3^Imaging Science-Based Lung and Bone Diseases Research Center, Wonkwang University, Iksan, Jeonbuk 570-749, Republic of Korea; ^4^Department of Radiology, School of Medicine, Wonkwang University, Iksan, Jeonbuk 570-749, Republic of Korea; ^5^Division of Rheumatology, Department of Internal Medicine, School of Medicine, Wonkwang University, 344-2 Sinyong-dong, Iksan, Jeonbuk 570-749, Republic of Korea; ^6^Institute for Skeletal Disease, Wonkwang University, Iksan, Jeonbuk 570-749, Republic of Korea

## Abstract

The risk of bone-related diseases increases due to the imbalance between bone resorption and bone formation by osteoclasts and osteoblasts, respectively. The goal in the development of antiosteoporotic treatments is an agent that will improve bone through simultaneous osteoblast stimulation and osteoclast inhibition without undesirable side effects. To achieve this goal, numerous studies have been performed to identify novel approaches using natural oriental herbs to treat bone metabolic diseases. In the present study, we investigated the effect of *Chrysanthemum indicum* extract (CIE) on the differentiation of osteoclastic and osteoblastic cells. CIE inhibited the formation of TRAP-positive mature osteoclasts and of filamentous-actin rings and disrupted the bone-resorbing activity of mature osteoclasts in a dose-dependent manner. CIE strongly inhibited Akt, GSK3*β*, and I*κ*B phosphorylation in RANKL-stimulated bone marrow macrophages and did not show any effects on MAP kinases, including p38, ERK, and JNK. Interestingly, CIE also enhanced primary osteoblast differentiation via upregulation of the expression of alkaline phosphatase and the level of extracellular calcium concentrations during the early and terminal stages of differentiation, respectively. Our results revealed that CIE could have a potential therapeutic role in bone-related disorders through its dual effects on osteoclast and osteoblast differentiation.

## 1. Introduction

Osteoporosis is a major metabolic bone disease that has been shown to cause increased risk of fractures, particularly in postmenopausal women and aging populations [1, 2]. The pathogenic mechanisms associated with symptoms of osteoporosis are mainly regulated by two cell types, osteoclasts and osteoblasts. The balanced interaction of osteoclast-specific bone resorptive action and osteoblast-specific bone formative action is crucial for maintenance of normal skeletal conditions [3]. However, an increased relative ratio of bone dissolving activity, caused by diverse factors, including the upregulation of intracellular reactive oxygen species (ROS) and oestrogen deficiency, has been shown to attenuate bone mass and results in various bone-related disorders such as osteoporosis, rheumatoid arthritis, and periodontitis [4–6].

Hematopoietic precursor cells of the macrophage/monocyte lineage differentiate into multinucleated osteoclasts in the presence of macrophage colony-stimulating factor (M-CSF) and receptor activator of NF-*κ*B ligand (RANKL), which are two key cytokines for regulating osteoclast survival and differentiation [7, 8]. During the process of RANKL-induced osteoclastogenesis, tumour necrosis factor receptor-associated factor 6 (TRAF6) is initially recruited through the interaction of RANKL and its receptor, RANK [9]. Subsequently, the activation of downstream signalling cascades of the RANKL-dependent pathway, including mitogen-activated protein kinases (MAPKs) comprised of p38, c-Jun N-terminal kinase (JNK), extracellular signal-regulated kinase (ERK), and Akt, by NF-*κ*B, is followed by the nuclear translocation of two transcription factors, c-Fos and nuclear factor of activated T cell c1 (NFATc1), which act as a binding agent in the promoter sites of its target genes. Thereafter, various osteoclast-marker genes, including osteoclast-associated receptor (*OSCAR*), tartrate-resistant acid phosphatase (*TRAP*), and* cathepsin K,* are finally activated to affect the formation and function of mature osteoclasts [10–12].

Osteoblasts, which are one of the key cell types in the skeleton, are derived from mesenchymal stem cells. During the process of osteogenesis, alkaline phosphatase (ALP) is released as an early-stage indicator of bone formative activity, and the level of extracellular matrix mineralization (ECM) increases during later stages [13, 14]. The activation of several transcription factors such as runt-related transcription factor 2 (Runx2), osterix, and *β*-catenin is required to regulate osteoblast differentiation [15].* Runx2 *plays a critical role in osteoblastogenesis through the control of cell proliferation and differentiation [16]. During the early stages of differentiation,* Runx2* expression is inversely correlated to the proliferation of osteoprogenitors and committed osteoblasts [17], and it is crucial to drive their transition to mature osteoblasts.* Runx2* binds osteoblast-specific* cis*-acting element 2 (OSE2) DNA sequences which is initially identified to locate in the 147th base pair of the osteocalcin promoter and is present in the promoter of other several genes, such as type I collagen alpha 1 (*Col1* *α* ) or osteopontin (*OPN*), to promote their expression and the establishment of the osteoblast phenotype [18, 19]. These extracellular proteins comprise the unique portion of the bone matrix that mineralizes in the presence of calcium phosphate crystals. Later,* Runx2* expression is downregulated to achieve a fully mature osteoblast phenotype [16].

Recently, numerous studies have been performed to investigate whether various natural extracts or representative compounds from oriental herbs exhibit a dual effect by inhibiting osteoclast differentiation, in addition to enhancing osteoblast differentiation, in order to identify novel therapeutic materials for treating bone disorders such as osteoporosis and rheumatoid arthritis [20–23]. To discover new extracts or functional food that can act as antiosteoporosis agent that promotes bone anabolic activity and inhibits osteoclast differentiation, we screened 20 natural extracts using analysis of* TRAP* staining and* ALP* staining and found that* Chrysanthemum indicum* has stimulatory effects on osteoblast differentiation and inhibitory effects on osteoclast differentiation.* Chrysanthemum indicum* is an oriental herb that has been widely used as a traditional Korean medicine to treat inflammation-related diseases. Numerous previous studies have demonstrated the pharmacological effects of* Chrysanthemum indicum *ethanol extracts (CIE) on hepatotoxicity, hypertension, and respiratory disorders [24, 25]. Also, CIE has suppression effects on prostate cancer which is proved to induce low bone density phenomenon and high risk of fracture, as symptoms of osteoporosis [26, 27]. However, there is no evidence that CIE prevents bone-related diseases, such as osteoporosis. In particular, the effect of CIE on osteoclast/osteoblast differentiation and the molecular mechanism underlying the therapeutic effects of CIE on osteoclastogenesis have not yet been identified.

In this study, we attempted to demonstrate the beneficial effects of CIE on RANKL-induced osteoclast differentiation as well as the effects of CIE to promote osteoblast differentiation. In addition, the inhibitory effect of CIE on bone-resorbing activity was examined to verify the value of CIE as a therapeutic agent for mediating bone diseases such as osteoporosis, rheumatoid arthritis, and osteopenia.

## 2. Materials and Methods

### 2.1. Reagents

A 95% CIE solution was purchased from the Korean Plant Extract Bank (Daejeon, Korea).* TRAP* staining solution and *β*-actin (housekeeping gene) were obtained from Sigma-Aldrich (St. Louis, MO, USA), and a sodium 3′-[1-(phenyl-aminocarbonyl)-3,4-tetrazolium]-bis(4-methoxy-6-nitro) (XTT) assay kit was purchased from Roche (Indianapolis, IN, USA). *α*-Minimum essential medium (*α*-MEM), foetal bovine serum (FBS), and penicillin-streptomycin were purchased from Gibco-BRL (Grand Island, NY, USA), and soluble human recombinant M-CSF and RANKL were purchased from Peprotech (London, UK). Specific antibodies against c-Fos and NFATc1 were obtained from Santa Cruz Biotechnology (Santa Cruz, CA, USA). Specific primary antibodies against phospho-p38, p38, phospho-Akt, Akt, phospho-JNK, JNK, phospho-I*κ*B, I*κ*B, phospho-ERK, ERK, phospho-GSK3*β*, and GSK3*β* were purchased from Cell Signaling Technology (Beverly, MA, USA). All other chemicals were of analytical or cell-culture grade.

### 2.2. Mouse Bone Marrow Macrophage Isolation and Osteoclast Differentiation

Mouse bone marrow cells were obtained from the femurs and tibiae of 5-week-old ICR mice and were incubated in *α*-MEM containing 10% FBS, 1% penicillin/streptomycin, and M-CSF (10 ng/mL) for 1 day to obtain nonadherent cells. The nonadherent cells, designated as osteoclast precursors, were incubated in *α*-MEM containing 10% FBS, 1% penicillin/streptomycin, and M-CSF (30 ng/mL) for 3 days. After 3 days, the resulting adherent cells that were obtained were used as bone marrow macrophages (BMMs). BMMs were incubated with M-CSF (30 ng/mL) and RANKL (100 ng/mL) in the presence of CIE (5–50 *μ*g/mL) or dimethyl sulfoxide (DMSO) as the control condition. After 3 days, the culture medium was refreshed with the same specified medium. On the following day, cells were fixed with 3.7% formalin for 20 min and permeabilized with 0.1% Triton X-100. The cells were then stained with* TRAP *solution and the stained cells were counted to determine the level of osteoclast differentiation.

### 2.3. Primary Osteoblast Cell Culture and Assays

Calvarial osteoblasts were cultured by the method described previously [22]. In short, the calvaria of 1-day-old mice was digested with 0.1% collagenase and 0.2% dispase 5 times and cultured in 10% *α*-MEM. For* ALP *staining, primary osteoblasts were cultured in the presence of 50 mg/mL ascorbic acid and 10 mM *β*-glycerol phosphate. After 7 days of differentiation, cells were fixed in 70% ethanol and stained for 20 min with a solution containing 0.01% naphthol, AS-MX phosphate, 1% N,N-dimethyl formamide, and 0.06% fast blue BB (Sigma). To analyse* ALP* activity after 7 days of differentiation, the cells were dissolved in 50 mM Tris-HCl buffer (pH 7.4) containing 1% Triton X-100, 150 mM NaCl, and 1 mM ethylenediaminetetraacetic acid (EDTA). Then, 100 mL of substrate (*p*-nitrophenylphosphate) (Sigma) was added to the cells, and the plate was incubated for 30 min at 37°C. The amount of* p*-nitrophenylphosphate was analysed by measuring the sample absorbance at 405 nm using a microplate reader. After 21 days of differentiation, cells were stained using Alizarin red S (ARS). The cultured cells were fixed in 3.7% formalin and stained for 20 min with a 2% ARS (Sigma) solution. To analyse ARS activity, the ARS in stained cells was dissolved by incubating the cells in a 10% cetylpyridinium chloride monohydrate (CPC) solution (Sigma) for 2 h with shaking. The absorbance was then measured at 545 nm using an enzyme-linked immunosorbent assay (ELISA) reader (Molecular Devices, CA, USA).

### 2.4. Coculture System of BMCs with Calvarial Primary Osteoblasts

BMCs (3 × 10^4^ cells/well) and primary osteoblasts (3 × 10^5^ cells/well) were cocultured with or without CIE (50 *μ*g/mL) for 5 days in the presence of IL-1 (10 ng/mL) in 48-well plates. After that, mature osteoclasts induced by coculture system were fixed with 3.7% formalin for 20 min and permeabilized with 0.1% Triton X-100. The cells were then stained with* TRAP *solution and the stained cells were counted to determine the level of osteoclast differentiation in the coculture system.

### 2.5. Cell Viability Assay

BMMs (1 × 10^4^ cells/well) were cultured with or without CIE (5–50 *μ*g/mL) for 3 days in the presence of M-CSF (30 ng/mL) in 96-well plates. Cells were then incubated for 4 h in a medium containing 50 *μ*L of XTT solution, benzenesulfonic acid hydrate, and N-methyl dibenzopyrazine methyl sulphate, and the optical density was read at 450 nm using an ELISA reader (Molecular Devices).

### 2.6. Western Blot Analysis

BMMs were lysed in a lysis buffer containing 50 mM Tris-HCl, 150 mM NaCl, 5 mM EDTA, 1% Triton X-100, 1 mM sodium fluoride, 1 mM sodium vanadate, 1% deoxycholate, and protease inhibitors. The lysate was centrifuged at 14,000 rpm for 20 min to obtain pure protein. The protein concentration was measured using a Bio-Rad colorimetric protein assay kit (Bio-Rad Laboratories Inc., Hercules, CA, USA), and equal amounts of proteins were separated with a sodium dodecyl sulphate-polyacrylamide gel. The proteins were transferred to a polyvinylidene fluoride membrane (Millipore, Bedford, MA, USA) and treated with 5% nonfat dry milk to inhibit attachment of nonspecific proteins. After the membrane was treated with primary and secondary antibodies (horseradish peroxidase-conjugated sheep anti-mouse or donkey anti-rabbit immunoglobulin), expression of specific protein signals was measured using a chemiluminescence detection system (Millipore).

### 2.7. Quantitative Real-Time Polymerase Chain Reaction (PCR) Analysis

Total RNA was extracted using QIAzol lysis reagent (Qiagen, Valencia, CA, USA) according to the manufacturer's instructions, and equal amounts of cDNA from the RNA samples were synthesized using 1 *μ*g of total RNA and SuperScript II Reverse Transcriptase (Invitrogen, San Diego, CA, USA). Real-time PCR was performed using an Exicycler 96 Real-Time Quantitative Thermal Block (Bioneer Co., Daejeon, Korea) with a 20 *μ*L reaction mixture containing 10 *μ*L SYBR Green Premix (Bioneer Co.), 10 pmol forward primer, 10 pmol reverse primer, and 1 *μ*g cDNA. The real-time PCR protocol consisted of the following steps: initial denaturation at 95°C for 5 min and 40 cycles of 3 PCR steps (denaturation at 95°C for 1 min, annealing at 60°C for 30 s, and extension at 72°C for 1 min). Gene expression was normalized to the housekeeping gene, glyceraldehyde 3-phosphate dehydrogenase (*GAPDH*). Relative results for specific genes were calculated using the comparative cycle threshold method. Real-time PCR was performed using the following primer sets: c-Fos, forward 5′-CTGGTGCAGCCCACTCTGGTC-3′ and reverse 5′-CTTTCAGCAGATTGGCAATCTC-3′; NFATc1, forward 5′-CAACGCCCTGACCACCGATAG-3′ and reverse 5′-GGCTGCCTTCCGTCTCATAGT-3′;* TRAP*, forward 5′-ACTTCCCCAGCCCTTACTAC-3′ and reverse 5′-TCAGCACATAGCCCACACCG-3′;* OSCAR*, forward 5′-CTGCTGGTAACGGATCAGCTCCCCAGA-3′ and reverse 5′-CCAAGGAGCCAGAACCTTGGAAACT-3′;* cathepsin K*, forward 5′-CACTGCTCTCTTCAGGGCTT-3′ and reverse 5′-ACGGAGGCATTGACTCTGAA-3′;* DC-STAMP*, forward 5′-GCAAGGAACCCAAGGAGTCG-3′ and reverse 5′-CAGTTGGCCCAGAAAGAGGG-3′;* OC-STAMP*, forward 5′-TGGGCCTCCATATGACCTCGAGTAG-3′ and reverse 5′-TCAAAGGCTTGTAAATTGGAGGAGT-3′;* integrin* *α* *ν* , forward 5′-TTGTTGCCGCCTTACGAGAA-3′ and reverse 5′-GCAGATGGCATAGCCACAGG-3′;* integrin *β*3*, forward 5′-TCTCCTGCGTCCGCTACAAA-3′ and 5′-CCCTTGGGACACTCAGGCTC-3′;* ICAM-1*, forward 5′-AGGCCACCCCAGAGGACAAC-3′ and reverse 5′-CCCATTATGACTGCGGCTGCTA-3′;* OPN*, forward 5′-TCTGATGAGACCGTCACTGC-3′ and reverse 5′-CCTCAGTCCATAAGCCAAGC-3′;* ALP*, forward 5′-GCTGATCATTCCCACGTTTT-3′ and reverse 5′-ACCATATAGGATGGCCGTGA-3′;* Col1* *α* , forward 5′-TGTGTTCCCTACTCAGCCGTCT-3′ and reverse 5′-CATCGGTCATGCTCTCTCCAA-3′;* Runx2*, forward 5′-TGCCTTCAGCACCCTATACC-3′ and reverse 5′-AGGTTGGAGGCACACATAGG-3′;* RANKL*, forward 5′-GACTCCATGAAAACGCAGGT-3′ and reverse 5′-TGTGTTGCAGTTCCTTCTGC-3′;* osteoprotegerin* (*OPG*), forward 5′-ACCTCACCACAGAGCAGCTT-3′ and reverse 5′-GCTCGATTTGCAGGTCTTTC-3′; and* GAPDH*, forward 5′-ACCACAGTCCATGCCATCAC-3′ and reverse 5′-TCCACCACCCTGTTGCTGTA-3′.

### 2.8. Bone Resorption Assay

Osteoclast pit assay is based on the previous report [23]. Briefly, BMCs and primary osteoblasts were cocultured in collagen gel-coated culture dishes for 7 days in the presence of 1,25-dihydroxyvitamin D_3_ (VitD_3_) (Sigma) and prostaglandin E_2_ (PGE_2_) (Sigma). After 7 days, the cocultured cells were detached by incubating in 0.1% collagenase at 37°C for 10 min and the separated cells were replated on hydroxyapatite-coated plates (Corning, NY, USA) with or without CIE (50 *μ*g/mL) for 24 h. After incubation, the cells on the plates were removed and the resorption pits were photographed and quantified using Image-Pro Plus software version 4.0 (Media Cybernetics; Silver Spring, MD, USA).

### 2.9. Filamentous-Actin Assay

BMMs were incubated with M-CSF (30 ng/mL) and RANKL (100 ng/mL) in the presence of CIE (5–50 *μ*g/mL) or DMSO as the control condition. After 3 days, the culture medium was replenished. On the following day, the cells were fixed in 3.7% formalin for 20 min and permeabilized with 0.1% Triton X-100 for 15 min. The cells were adapted in 0.25% bovine serum albumin (BSA) for 30 min, and then the cells were stained with phalloidin and DAPI solution (Life Technologies, Carlsbad, CA, USA).

### 2.10. Retroviral Gene Transfection

The retroviral vectors pMX-IRES-EGFP, pMX-cFos-IRES-EGFP, and pMX-NFATc1-IRES-EGFP packaging were performed by transient transfection of these pMX vectors into Plat-E retroviral packaging cells using X-tremeGENE 9 (Roche, Nutley, NJ, USA) according to the manufacturer's instructions. After incubation in fresh medium for 2 days, the culture supernatants of the retrovirus-producing cells were collected. For retroviral infection, nonadherent BMCs were cultured in M-CSF (30 ng/mL) for 2 days. The BMMs were incubated with viral supernatant pMX-IRES-EGFP, pMX-cFos-IRES-EGFP, and pMX-NFATc1-IRES-EGFP virus-producing Plat-E cells together with polybrene (10 *μ*g/mL) and M-CSF (30 ng/mL) for 6 h. The infection efficiency of the retrovirus was determined by green fluorescent protein expression and was consistently >80%. After infection, the BMMs were induced to differentiate in the presence of M-CSF (30 ng/mL) and RANKL (100 ng/mL) for 4 days. The forced expression of each construct and osteoclast formation was detected using a fluorescence microscope and* TRAP *staining.

### 2.11. Statistical Analyses

Experiments were conducted separately at least 3 times and all data are presented as the mean ± standard deviation (SD). All statistical analyses were performed using SPSS software (Korean version 14.0). The statistical differences were analysed using one-way ANOVA followed by a Tukey post hoc test. *P* values less than 0.05 were considered statistically significant.

## 3. Results

### 3.1. CIE Inhibits Osteoclast Formation in Bone Marrow Cells-Osteoblast Cocultures and Primary BMMs

**Figure 1:**
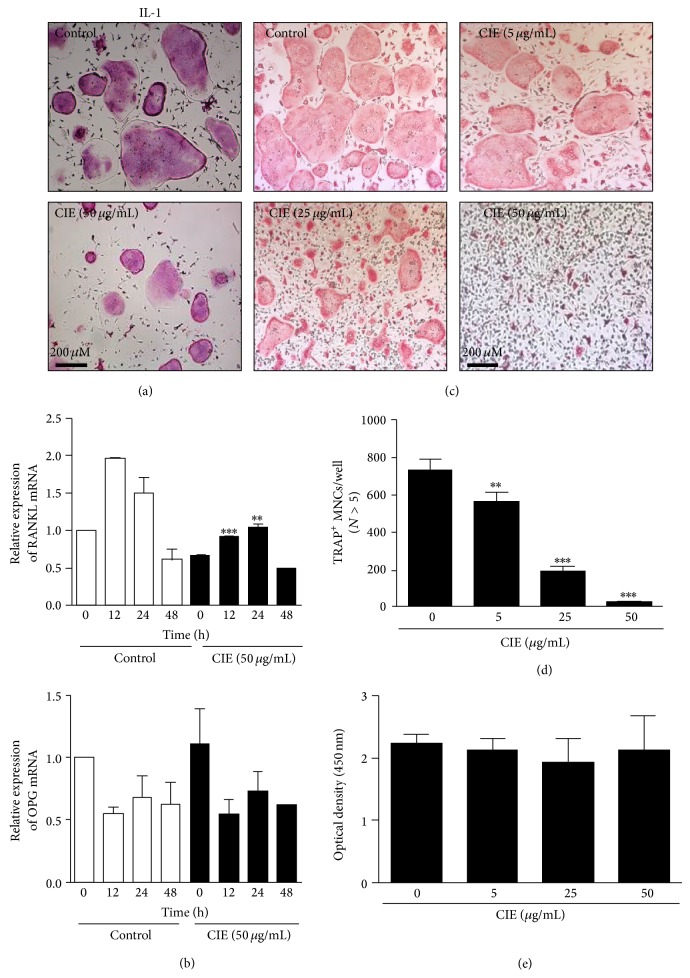
CIE inhibits the formation of TRAP-positive osteoclasts from cocultures and BMMs. (a) Mouse bone marrow cells and primary osteoblasts were cocultured with IL-1 (10 ng/mL) in the presence of CIE (50 *μ*g/mL) or DMSO (control) for 5 days. Cells were fixed with 3.7% formalin in PBS, permeabilized with 0.1% Triton X-100 in PBS, and stained with* TRAP* solution. (b) Mouse primary osteoblasts were pretreated with CIE (50 *μ*g/mL) or DMSO (control) for 1 h and then were stimulated with IL-1 (10 ng/mL) for indicated times. The expressions of RANKL and OPG were determined by using real-time RT-PCR analysis. (c) BMMs were cultured for 4 days in the presence of M-CSF (30 ng/mL) and RANKL (100 ng/mL) with either DMSO or CIE. Cells were detected by* TRAP *staining. (d) TRAP-positive multinucleated cells were counted as osteoclasts. _ _^**^ *P* < 0.01; _ _^***^ *P* < 0.001. (e) BMMs were cultured for 3 days with the indicated doses of CIE in the presence of M-CSF (30 ng/mL). Cell viability was analysed using an XTT assay.

We investigated the effect of CIE on osteoclast formation induced by osteoclastogenic factors in cocultures of bone marrow cells and primary osteoblast* in vitro*. Stimulation of IL-1 for 5 days formed TRAP-positive multinucleated osteoclasts in the cocultures and, under these conditions, treatment with CIE significantly inhibited osteoclast formation (Figure 1(a)). In these culture systems, osteoblasts support osteoclastogenesis from osteoclast precursors by regulating the expression of RANKL and OPG [28]. We thus evaluated by RT-PCR whether CIE affects the expression of RANKL and OPG in osteoblasts. The addition of osteoclastogenic factor, IL-1, increased RANKL expression and decreased OPG expression at 24 h in osteoblasts. Treatment with CIE suppressed the IL-1-induced RANKL expression, with no marked change in OPG expression (Figure 1(b)). We next examined the effects of CIE on RANKL-induced osteoclastogenesis from osteoclast precursor BMMs. The formation of TRAP-positive multinucleated cells was distinctly apparent in BMMs treated with DMSO (as a control) in the presence of M-CSF and RANKL. However, the differentiation of BMMs pretreated with CIE into osteoclasts was limited in a dose-dependent manner (Figure 1(c)). In addition, we found that the number of TRAP-positive osteoclasts was dramatically decreased (Figure 1(d)). Next, we used an XTT assay to determine if the potential inhibitory effect of CIE on osteoclastogenesis was associated with cytotoxicity. At the evaluated concentrations CIE did not affect cell viability, even at high concentrations (Figure 1(e)).

### 3.2. CIE Attenuates RANKL-Mediated Induction of c-Fos and NFATc1

**Figure 2:**
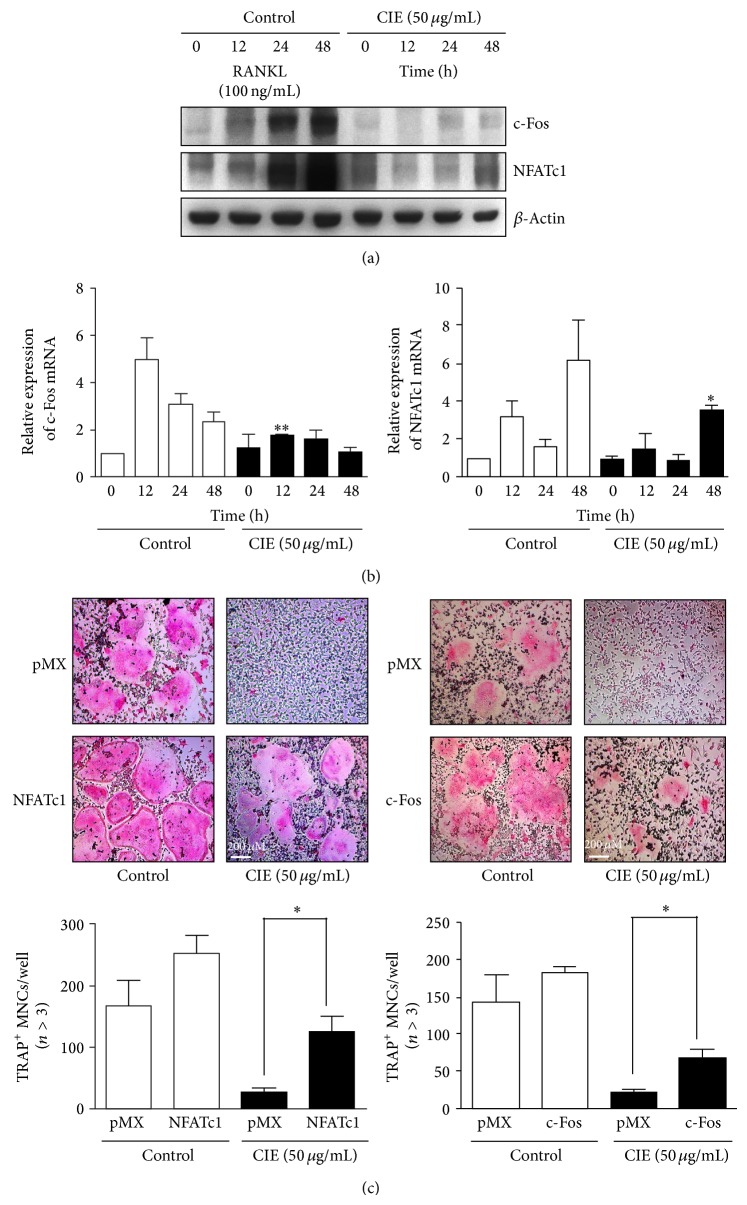
CIE suppresses RANKL-induced c-Fos and NFATc1 expression. (a) Effects of CIE on levels of c-Fos and NFATc1 protein expression were evaluated using western blot analysis. *β*-Actin was used as the internal control. (b) BMMs were stimulated with RANKL (100 ng/mL) and M-CSF (30 ng/mL) in the presence or absence of CIE (50 *μ*g/mL) for the specified times. Total RNA was isolated from cells using QIAzol reagent and mRNA expression levels of c-Fos and NFATc1 were evaluated using real-time PCR. _ _^*^ *P* < 0.05 versus the control at 48 h; _ _^**^ *P* < 0.01 versus the control at 12 h. (c) BMMs were infected with retroviruses expressing pMX-IRES-EGFP (pMX), pMX-NFATc1-EGFP, or pMX-cFos-EGFP. Infected BMMs were cultured with or without CIE (50 *μ*g/mL) in the presence of M-CSF (30 ng/mL) and RANKL (100 ng/mL) for 4 days. After culturing, the cells were fixed and stained for* TRAP *(top). The TRAP-positive multinucleated osteoclasts were counted (bottom). _ _^*^ *P* < 0.05.

In order todetermine whether CIE downregulates c-Fos and NFATc1, two key modulators of osteoclastogenesis, we examined the effects of CIE on the mRNA or protein levels of c-Fos and NFATc1 using quantitative real-time PCR or western blot analysis, respectively. RANKL-induced expression of c-Fos and NFATc1 was significantly reduced in the presence of CIE (Figures 2(a) and 2(b)). In addition, to confirm the possibility that the expression of c-Fos or NFATc1 is sufficient to reverse the effects of CIE on osteoclastogenesis, we applied a retrovirus overexpressing c-Fos or CA-NFATc1. As shown in Figure 2(c), the infection of* c-Fos* or* CA-*NFATc1 rescued the inhibitory effect of CIE on osteoclast differentiation, suggesting that CIE induces inactivation of c-Fos and NFATc1.

### 3.3. CIE Regulates Osteoclastogenesis by Suppressing Early Signalling and the Expression of Specific Genes

**Figure 3:**
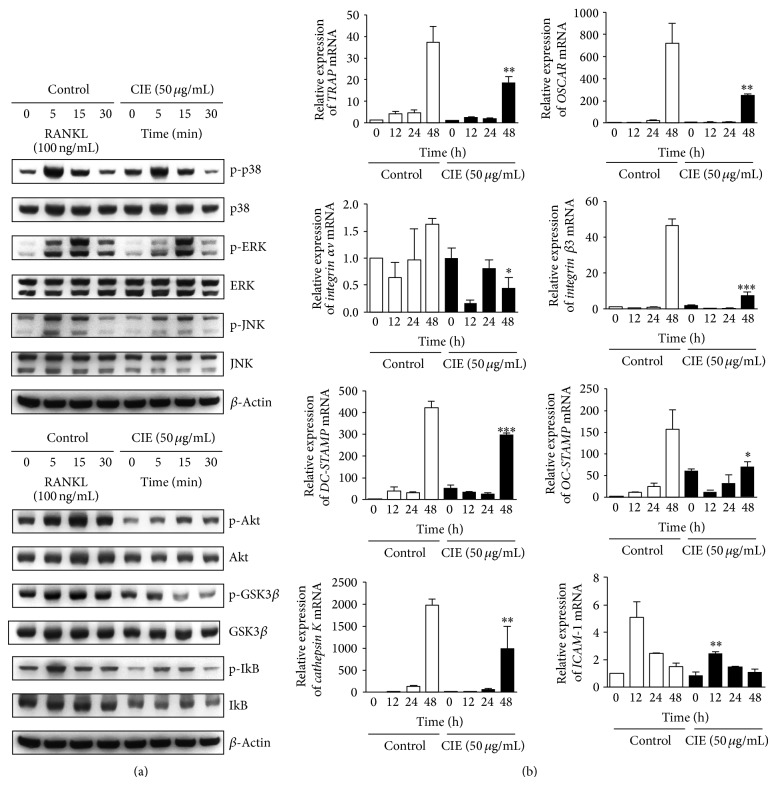
CIE downregulates RANKL-induced early signals and marker genes during osteoclastogenesis. (a) BMMs were pretreated with DMSO (control) or CIE (50 *μ*g/mL) for 1 h in the presence of M-CSF (30 ng/mL) and were stimulated with RANKL (100 ng/mL) for the indicated times. Whole-cell lysates were used for western blot analysis with the specified antibodies. *β*-Actin served as the internal control. (b) BMMs were stimulated with RANKL (100 ng/mL) and M-CSF (30 ng/mL) in the presence or absence of CIE (50 *μ*g/mL) for the indicated times. Total RNA was isolated from cells using QIAzol reagent and the mRNA expression levels of* OSCAR, TRAP, integrin *α*v, *β*3, DC-STAMP, OC-STAMP, cathepsin K,* and* ICAM-1* were evaluated by real-time PCR. _ _^*^ *P* < 0.05, _ _^**^ *P* < 0.01, and _ _^***^ *P* < 0.001 versus the control at 12 h, 24 h, and 48 h.

To elucidate the inhibitory mechanism and pathways influenced by CIE, we evaluated the effect of CIE on MAP kinases, including p38, ERK, JNK, and I*κ*B, Akt, and the GSK3*β* pathway, which are widely known to be essential for osteoclast differentiation. As shown in Figure 3(a), RANKL-induced phosphorylation of MAP kinases was not affected by treatment with CIE. However, the phosphorylation of Akt, GSK3*β*, and I*κ*B and the degradation of I*κ*B by RANKL were decreased by treatment with CIE. In addition, we also analysed the effect of CIE on various genes related to osteoclast formation and function at the mRNA level. CIE downregulated the expression of* OSCAR* and* TRAP*, which are genes specifically related to osteoclast formation. Moreover, CIE also suppressed* DC-STAMP*,* OC-STAMP*,* ICAM-1,* and* integrin *α*v*β*3 *expression, which are known to affect cell-cell interactions such as migration or fusion. Expression of* cathepsin K*, which is related to bone-resorbing activity, was also significantly inhibited (Figure 3(b)). These results indicate that the inhibitory effects of CIE on RANKL-mediated NF-*κ*B, Akt, and GSK3*β* signalling are followed by the inactivation of osteoclast-specific marker genes.

### 3.4. CIE Blocks the Formation of Actin Rings and the Bone-Resorbing Activity of Mature Osteoclasts in a Dose-Dependent Manner

**Figure 4:**
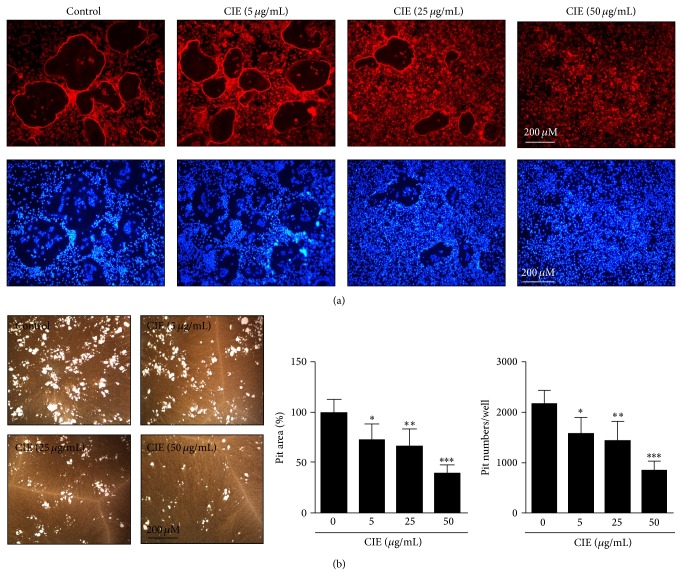
CIE blocks actin ring formation and the bone-resorbing activity of mature osteoclasts. (a) BMMs were cultured for 4 days in the presence of M-CSF (30 ng/mL) and RANKL (100 ng/mL) with DMSO (control) or various concentrations of CIE. Cells were fixed with 3.7% formalin in PBS, permeabilized with 0.1% Triton X-100 in PBS, and stained with phalloidin and DAPI. (b) Mature osteoclasts were seeded on hydroxyapatite-coated plates for 24 h with the indicated concentrations of CIE. Attached cells on the plates were removed and photographed under a light microscope. The relative ratio and number of pit areas were quantified using Image J. _ _^*^ *P* < 0.05, _ _^**^ *P* < 0.01, and _ _^***^ *P* < 0.001.

We cultured primary BMMs treated with various concentrations of CIE or DMSO to determine whether CIE regulates filamentous-actin (F-actin) ring formation. The formation of actin rings was distinctly apparent in BMMs treated with DMSO (as a control). However, the differentiation of BMMs treated with CIE into mature osteoclasts with F-actin structure was limited in a dose-dependent manner (Figure 4(a)). We seeded mature osteoclasts on top of the hydroxyapatite-coated plate with or without CIE. While considerable resorption pit formation was observed in the DMSO control condition, the formation of resorption pit areas in the hydroxyapatite-coated plate was suppressed in mature osteoclasts treated with CIE. In addition, the relative ratio and number of pit areas were decreased dose-dependently (Figure 4(b)). These results suggest that CIE suppresses the formation of actin ring structures and the bone matrix-dissolving activity of mature osteoclasts.

### 3.5. CIE Enhances Primary Osteoblast Differentiation and mRNA Expression of Osteoblast-Specific Gene Markers

**Figure 5:**
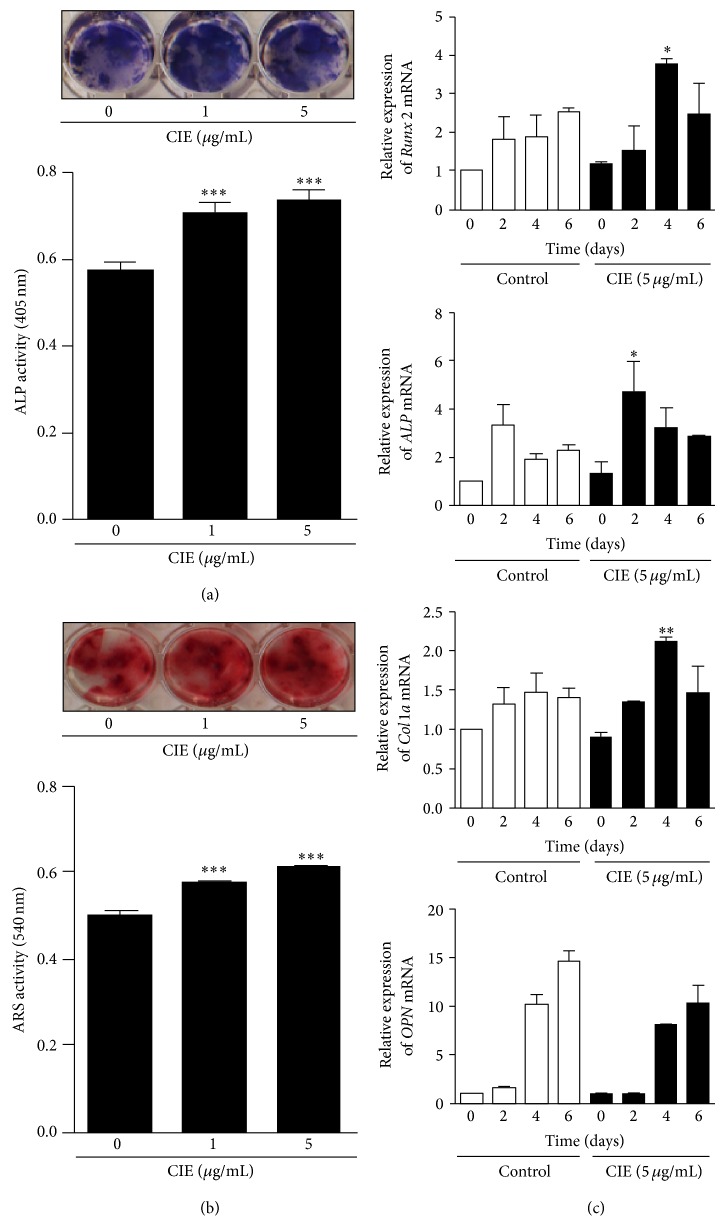
CIE promotes ascorbic acid and *β*-glycerol phosphate mediated osteoblast differentiation. (a) Primary osteoblasts were treated with various concentrations of CIE for 7 days in the presence of 50 *μ*g/mL ascorbic acid and 10 mM *β*-glycerol phosphate. ALP-positive cells were stained with* ALP *solution. (b) Primary osteoblasts were treated with various concentrations of CIE for 21 days. Calcium accumulation within the osteoblasts was stained with ARS solution. Stained calcium deposits were dissolved by 10% CPC buffer to measure the level of staining. (c) Primary osteoblasts were stimulated with ascorbic acid (50 *μ*g/mL) and *β*-glycerol phosphate in the presence of CIE (50 *μ*g/mL) or DMSO. Total RNA was isolated from cells using QIAzol reagent and mRNA expression levels of* Runx2, ALP, Col1*α*, *and* OPN *were evaluated by real-time PCR. _ _^*^ *P* < 0.05, _ _^**^ *P* < 0.01, and _ _^***^ *P* < 0.001.

To identify the effect of CIE on early-stage osteoblast differentiation, we examined* ALP* staining of primary osteoblasts. As shown in Figure 5(a), CIE increased the level of* ALP* expression and* ALP *activity dose-dependently. In addition, to examine the effect of CIE-induced mineralization during osteoblastogenesis, ARS staining in osteoblasts was assessed after 21 days of culture. In differentiated osteoblasts without CIE, the ECM calcium deposition in the mineralized matrix was minimal. There was substantial ARS staining in cells differentiated with CIE for 21 days, which increased with CIE treatment. These results showed that CIE enhances initial osteoblast differentiation by increasing* ALP *activity and enhances terminal osteoblast differentiation by increasing mineralization. As shown in Figure 5(a), the level and intensity of ARS staining, indicative of ECM mineralization, were increased by treatment with CIE. These results demonstrate that CIE affects both the initial and terminal stages of osteoblast differentiation. Next, we examined the expression of genes related to osteoblast differentiation. The expressions of* Runx2*,* ALP*, and* ColIa* were increased by treatment with CIE in comparison to the DMSO-treated control group. However, CIE did not stimulate the expression of* OPN* (Figure 5(b)).

## 4. Discussion

In the present study, we found that CIE prevents IL-1-induced RANKL induction in osteoblasts and osteoclast differentiation from bone marrow cells and primary osteoblast cocultures. Many osteoclastogenic factors including IL-1, TNF-*α*, and PGE_2_ plus VitD_3_ induce osteoclast differentiation by upregulation of RANKL/OPG ratio. In particular, IL-1 is thought to be a critical mediator of the bone loss induced by estrogen deficiency and inflammation [29, 30]. IL-1 increases RANKL expression in osteoblasts by stimulating PGE_2_ production through cytosolic phospholipase A2 (cPLA2) and COX2-dependent mechanisms [31]. Thus, these reports suggest that the inhibition of RANKL expression by CIE in osteoblast is, as least in part, involved in the reduced PGE_2_ synthesis. We also found that CIE has direct inhibitory effects on both RANKL-induced osteoclastogenesis in BMMs and the functional activity of giant osteoclasts as well as indirect effects via osteoblasts. Furthermore, we demonstrated the stimulatory effect of CIE on osteoblastic differentiation of primary osteoblast cells by measuring the levels of* ALP *and calcium deposits.

Osteoclastic bone resorption is a multistep process that involves commitment from hematopoietic progenitors, fusion of the cells, development of a ruffled border and a clear zone, and the secretion of acids and lysosomal enzymes into a resorbing area [12]. Our data suggest that CIE strongly inhibits the formation of an F-actin ring and bone resorption by mature osteoclasts, as well as osteoclast differentiation* in vitro* (Figures 1 and 4). The delineation of the cascade of events leading to bone resorption should provide a molecular basis for the development of novel and specific therapeutic agents for bone deficit conditions such as osteoporosis. Cell-cell fusion is required for the formation of multinucleated osteoclasts and giant cells, although the mechanisms that govern these processes are poorly understood. The cell-cell fusion of osteoclast and foreign body giant cells was previously shown to be completely abrogated in* DC-STAMP*- or* OC-STAMP*-deficient mice in which the bone-resorbing activity was significantly reduced [32–34]. Our data suggest that CIE has the capacity to interrupt the formation of multinuclear mature osteoclasts by cell-cell fusion of mononuclear macrophages through disturbance of the* OC-STAMP*- and* DC-STAMP*-dependent pathway (Figure 3(b)). Cell-cell fusion of osteoclasts has also been shown to be required for cytoskeleton reorganization to seal the resorbing area for bone resorption [10, 35]. Several proteolytic enzymes, including* TRAP*,* OSCAR*, and* cathepsin K*, have been shown to play important roles in degrading the organic bone matrix, and, of these proteolytic enzymes,* TRAP*,* OSCAR*, and* cathepsin K* have the highest levels in osteoclasts. Matrix-degrading enzymes are also known to be collagen-degrading enzymes, and they can directly degrade collagen in hard tissues that have been demineralized [36]. In addition, the*α* *ν* *β*3* integrin* is known to play a role in the regulation of cell migration and the maintenance of the sealing zone required for effective osteoclastic bone resorption [37]. In the present study, we found that CIE dampened cellular induction of* cathepsin K*,* integrin* *α* *ν* , and* integrin* *β*3 by RANKL, one of the sequential events leading to bone resorption. This indicates that CIE failed to induce cytoskeletal organization leading to the formation of a ruffled border and the adhesion of osteoclasts to the bone matrix.

Understanding the mechanistic basis of osteoclast function could provide a new therapeutic strategy for bone loss. Under normal conditions, the RANKL-RANK axis appears to be essential for osteoclastogenesis, and costimulatory immunoreceptors lead to robust induction of c-Fos and NFATc1 that are the necessary and sufficient transcription factors for osteoclast differentiation [9, 37, 38]. The crucial role of c-Fos in osteoclastogenesis was demonstrated by* in vivo* experiments using genetically modified mice, where c-Fos-deficient mice exhibited a severe osteoporotic phenotype due to the failure of osteoclast differentiation [39]. In addition, NFATc1-deficient embryonic stem cells were shown to be unable to differentiate into osteoclasts in response to RANKL, and the forced expression of NFATc1 has been shown to lead to the formation of osteoclasts from BMMs in the absence of RANKL [40]. In the present study, CIE inhibited the expression of c-Fos and NFATc1 in BMMs (Figure 2(a)). We further determined that the ectopic expression of c-Fos and NFATc1 is sufficient to rescue the inhibitory effect of CIE on the formation of TRAP-positive multinucleated cells (Figure 2(b)). Next, to gain insight into the molecular mechanism underlying CIE induced antiosteoclastogenic effects, we investigated the effects of CIE on RANKL-induced signalling pathways. Stimulation of RANKL has been reported to activate three well-known signalling pathways, including NF-*κ*B, MAP kinase, and PI3K/Akt/GSK3*β* [41, 42]. In the Akt signalling pathway, the deletion of Akt1, one of the Akt family members, induces impairment of bone-resorbing activity by reducing the level of RANKL in osteoblasts [43]. Moreover, the downregulation of the inactive form of GSK3*β*, due to the inhibition of Akt phosphorylation, blocks osteoclast formation via attenuating nuclear localization of NFATc1 [44]. Besides, activation of NF-*κ*B can be initiated by several different kinases such as Akt and NF-*κ*B inducing kinase. Gingery et al. demonstrated that Akt/NF-*κ*B axis is critical in osteoclastogenesis and maintaining mature osteoclast survival [45]. Our data show that CIE specifically downregulated RANKL-induced phosphorylation of Akt, GSK3*β*, and I*κ*B and the degradation of I*κ*B without affecting the activation of MAP kinase (Figure 3(a)). Thus, the antiosteoclastogenic action of CIE could be due to its potential to directly inhibit Akt signalling pathway that consequently downregulates the expression of NF-*κ*B and NFATc1.

Furthermore, CIE was shown to stimulate osteoblast differentiation. As shown in Figure 5, we confirmed the accelerating effect of CIE on the level of* ALP* and calcium phosphate mineral accumulation in primary osteoblasts by measuring* ALP *and ARS staining or activity. In addition, CIE enhanced osteoblastic biomarker genes such as* Runx2, ALP*, and *Col1* *α* .* Runx2* is a key regulator of chondroblast and osteoblast differentiation and of bone development* in vivo *[18, 46]. Targeted disruption of* Runx2* in mice was previously shown to result in a complete lack of ossification due to the arrest of osteoblast maturation [47, 48].* Runx2* regulates the expression of major ECM genes, including* ALP*,* Col1* *α*, and* OPN* [18]. Here, our data suggest that CIE-induced osteogenesis is due to enhanced* ALP* and* Col1* *α* gene expression via the* Runx2* transcription factor. However, the mechanisms underlying the stimulatory effect of CIE on osteoblastogenesis are not completely understood. Therefore, further investigation is needed to clarify how CIE influences osteoblast-specific transcription factors and various signalling pathways in the process of bone formation.

With this study, we still do not fully understand how CIE maintains dual specificity on bone remodelling. Nevertheless, we postulate that the downregulation of NF-*κ*B-, Akt-, and GSK3*β*-dependent signalling may be involved in the inhibitory effect of CIE on RANKL induction of c-Fos and NFATc1 during osteoclastogenesis and that the activation of* Runx2, ALP, *and* Col1* *α* is at least one of the critical steps necessary for the CIE-dependent increase in osteoblast differentiation.

Also,* in vivo* studies are further needed to verify the bone protective role of CIE. Previously,* in vivo* efficacy of CIE associated with various pharmacological actions has been elucidated. Oral administration of CIE could have hepatoprotective effect at 50 mg/kg and anti-inflammatory activity at 150 mg/kg without any side effects, such as toxicity [17, 18]. Presumably, consistent with the* in vitro* results, in reference to* in vivo* treatment dose of CIE, oral administration of CIE in the range of 50 to 150 mg/kg could suppress excessive bone resorption through downregulating RANKL expression of osteoblasts as well as interfering with molecular signaling of osteoclastogenesis. Simultaneously, CIE also could increase bone formation through enhancing osteoblastogenesis and expression of osteoblast marker genes. Accordingly,* in vivo* treatment with CIE is likely to regulate abnormally high bone turnover rate, leading to improvement of pathologic condition of osteoporosis.

## 5. Conclusion

Based on our results, we demonstrated that CIE regulates bone remodelling by inhibiting osteoclast differentiation and function and stimulating osteoblast function. This suggests the possibility for CIE as a novel dual-action therapeutic agent against osteoporosis and other bone diseases by regulating both bone resorption and formation.
